# Role of Probiotics and Diet in the Management of Neurological Diseases and Mood States: A Review

**DOI:** 10.3390/microorganisms10112268

**Published:** 2022-11-15

**Authors:** Subramanian Thangaleela, Bhagavathi Sundaram Sivamaruthi, Periyanaina Kesika, Chaiyavat Chaiyasut

**Affiliations:** 1 Innovation Center for Holistic Health, Nutraceuticals, and Cosmeceuticals, Faculty of Pharmacy, Chiang Mai University, Chiang Mai 50200, Thailand; 2 Office of Research Administration, Chiang Mai University, Chiang Mai 50200, Thailand

**Keywords:** probiotics, psychobiotics, cognition, Parkinson’s disease, Alzheimer’s disease, autism spectrum disorder, stress, anxiety, depression

## Abstract

Alzheimer’s (AD) and Parkinson’s diseases (PD) are common in older people. Autism spectrum disorders (ASD), anxiety, depression, stress, and cognitive impairment are prevalent among people irrespective of age. The incidence of neurological disorders has been increasing in recent decades. Communication between the gut microbiota and the brain is intrinsically complicated, and it is necessary for the maintenance of the gut, brain, and immune functions of the host. The bidirectional link among the gut, gut microbiota and the brain is designated as the “microbiota–gut–brain axis.” Gut microbiota modulates the host immune system and functions of tissue barriers such as gut mucosa and blood–brain barrier (BBB). Gut microbial dysfunction disturbs the gut–brain interplay and may contribute to various gut disorders, neurocognitive and psychiatric disorders. Probiotics could protect intestinal integrity, enhance gut functions, promote intestinal mucosal and BBB functions, and support the synthesis of brain-derived neurotrophic factors, which enhance neuronal survival and differentiation. Probiotics could be considered an adjunct therapy to manage metabolic and psychiatric diseases. Predominantly, *Lactobacillus* and *Bifidobacterium* strains are documented as potent probiotics, which help to maintain the bidirectional interactions between the gut and brain. The consumption of probiotics and probiotics containing fermented foods could improve the gut microbiota. The diet impacts gut microbiota, and a balanced diet could maintain the integrity of gut–brain communication by facilitating the production of neurotrophic factors and other neuropeptides. However, the beneficial effects of probiotics and diet might depend upon several factors, including strain, dosage, duration, age, host physiology, etc. This review summarizes the importance and involvement of probiotics and diet in neuroprotection and managing representative neurological disorders, injuries and mood states.

## 1. Introduction

Probiotics consumption is increasing globally because of their health benefits [1]. Probiotics are proven adjuvant therapeutic agents for various acute and chronic infections, cancer [2], inflammatory diseases [3], and cognitive and psychiatric disorders [4]. Approximately 20% of people worldwide suffer from mental health disorders such as depression and anxiety [5]. Psychologically good mental health can be described as the status of the individual’s well-being. Generally, psychological research carried out in rodents focuses on stress, anxiety, and motivation [6].

A panel of experts in the International Scientific Association for Probiotics and Prebiotics (ISAPP) officially described probiotics as “live microorganisms which, when administered in adequate amounts, confer a health benefit on the host” [7]. In 2013, a novel class of probiotics emerged as “psychobiotics.” Dinan and colleagues described psychobiotics as “live organisms that produce health benefits in psychiatric illness patients when ingested in adequate amounts” [8]. Sarkar and colleagues mentioned psychotropics and antibiotics for mental health disorders could be considered psychobiotics [6]. Psychobiotics work through synthesizing, distributing, and regulating the neurotransmitters such as gamma-aminobutyric acid (GABA), serotonin (5-HT), glutamate and brain-derived neurotrophic factor (BDNF) in executing and regulating the neural processes such as learning, memory, mood, and other cognitive functions [9].

Psychobiotics influence the central nervous system (CNS) through the gut–brain axis with the support of immune, neural, and metabolic pathways [9]. Psychobiotics are efficient in treating neurodegenerative and neurodevelopmental disorders by improving cognitive functions in Alzheimer’s’ disease (AD), motor functions in Parkinson’s disease (PD) and improve autism spectrum disorder (ASD) symptoms [9]. Many approaches have emerged to manage mental health through psychobiotics and dietary supplementation. The present manuscript review highlights the importance and benefits of psychobiotics and diet in managing neurological problems and mood states.

## 2. Methodology

**Figure 1:**
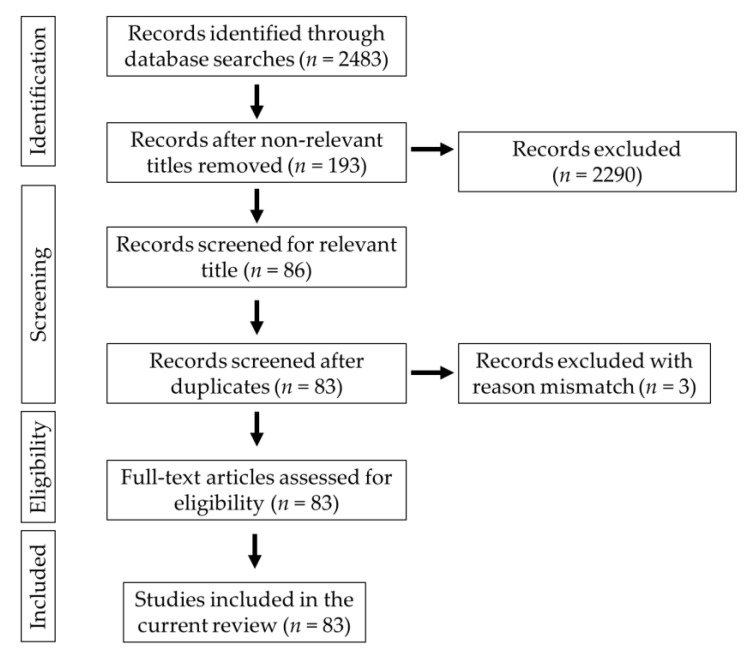
Schematic representation of the PRISMA chart explaining the selection of studies.

Keywords such as “gut microbiota–brain,” “gut–brain axis,” “probiotics and neurological diseases,” “Alzheimer’s disease,” “Parkinson’s disease,” “cognition,” “mood states,” “neurological injuries,” and “diet and brain” were used to search the documents in scientific databases such as PubMed, Google Scholar, Medline and PsycINFO. The relevant studies were selected for the preparation of the manuscript. The publications in the English language were used in the present study. The PRISMA (Preferred Reporting Items for Systematic Reviews and Meta-Analyses) chart explains the selection criteria of the collected articles (Figure 1).

## 3. Probiotics and Brain Health

Physical well-being is important for mental well-being and vice versa. Psychobiotics influence brain–bacteria communications through the enteric nervous system (ENS) and immune system and exert anxiolytic, anti-depressant properties resulting in enormous changes in the cognitive and emotional parameters [6]. Bacterial genera such as *Lactobacillus, Bifidobacterium, Enterococcus, Streptococcus* and *Escherichia* commonly influence the bidirectional interactions of the brain and gut system through the production of neurochemicals; they are used as psychobiotics. Unlike conventional probiotics, psychobiotics can synthesize or stimulate the production of various neurotransmitters, anti-inflammatory cytokines, and gastric endocrine hormones [10]. Balancing and maintaining an individual’s physical and emotional well-being requires two-way communication between the gut and brain, mainly regulated by the gut microbiota. Gut microbiota (GM) is important in neurodevelopmental and neuropsychiatric disorders [10].

The studies on psychobiotics and psychological illness are very limited. However, employing psychobiotics for treating neuropsychiatric diseases is growing as a new field of interest among neurological researchers: psychobiotics used in the treatment of stress, anxiety and depression and other mood disorders. It was found that psychobiotics reduced the cognitive reactivity to negative mood and improved depressive symptoms, anxiety, and stress response [11,12].

The microbial infection could affect mental processes, and the metabolically active multifaceted healthy intestinal microbiota provides positive mental health benefits [8]. Logan and Katzman first used probiotics as an adjunct therapy in managing major depressive disorder (MDD). In MDD, gut microflora becomes altered, and the levels of Lactobacilli and *Bifidobacterium* are lower, which could change the gut functions through elevated pro-inflammatory cytokines and oxidative stress. Probiotics could lower the pro-inflammatory cytokines, reduce oxidative stress, and increase BDNF [13].

GABA is the inhibitory neurotransmitter that plays a significant role in physiological and psychological functions. GABA receptors are important for normal behaviour. More specifically, GABA_B_ receptors play an important role in mood and anxiety disorders. Any changes in the expression of GABA receptors can cause anxiety and depression along with bowel disorders as comorbid conditions [14]. *Lactobacillus rhamnosus* (*L. rhamnosus*) reduced anxiety in stress-induced hyperthermia (SIH), elevated plus maze test (EPM) and forced swim test (FST) in mice. *L. rhamnosus* altered the expression of GABA_B1b_ receptors in the brain with an increase in cingulate and prelimbic regions and a decrease in the hippocampus, amygdala, and locus coeruleus. GABA_Aα2_ was reduced in the prefrontal cortex and amygdala and increased in the hippocampus compared to the control mice. *L. rhamnosus* reduced stress-induced corticosterone elevation and depression-related behaviours [14]. The results indicated that *L. rhamnosus* could change neural functions, which causes behavioural and neurological effects. Thus, it can be considered in therapeutic applications against depression.

Gut disorders with comorbid psychiatric conditions increase gut permeability, enhance lipopolysaccharide (LPS) translocation and increase depressive symptoms. *Lactobacillus farciminis* suppress acute psychological stress-induced gut permeability by attenuating the hypothalamus–pituitary–adrenal (HPA) axis in rats [15]. The oral administration of *Lactobacillus* increases the GABA, N-acetyl aspartate, and glutamate in the brain of mice [16]. *Bifidobacterium longum* (*B. longum*) 1714 enhanced the behaviour and cognitive performance in stressed mice [17]. Psychobiotics benefit neuropsychiatric disorders such as schizophrenia, Tourette’s syndrome, attention deficit hyperactivity disorder (ADHD), AD, PD, ASD, stress, depression, and anxiety [10]. In addition to neurodegenerative diseases, neurologic injuries such as traumatic brain injury (TBI), ischemic stroke, spinal cord injury (SCI), and haemorrhagic cerebrovascular lesions could cause gut dysbiosis. In another way, any changes in GM can produce proinflammatory cytokines and clotting factors, increasing the risk of neurological injuries [18].

**Figure 2:**
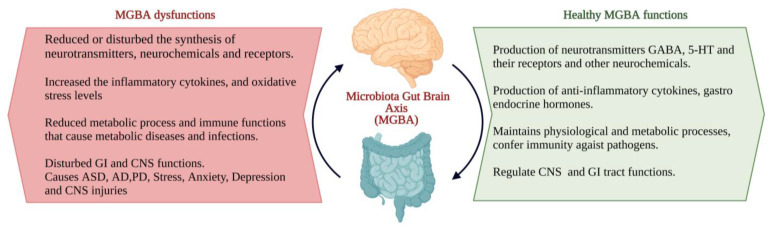
The characteristics of the ideal and consequents of dysfunctions of the microbiota–gut–brain axis (MGBA) [19,20,21,22]. (Figure created using BioRender.com; accessed on 17 October 2022.)

The interactions between the gut microbiota, gut and CNS are familiarly known as the microbiota–gut–brain axis (MGBA). Dysregulation in MGBA could cause intestinal disorders, stress, anxiety, depression, and other psychiatric disorders [14]. The emotional state depends on the gastrointestinal (GI) tract function. MGBA dysregulation can cause GI, neuropsychological, and metabolic disorders. The gut–brain interaction can be identified through the relationship between gut dysbiosis and GI and CNS disorders [19]. MGBA dysregulation increases intestinal permeability, promotes the proinflammatory phase and causes CNS injuries. The systemic inflammation might result in secondary CNS injuries [20]. High differentiation and migration of immune cells to the CNS can cause maladaptive CNS inflammation [21], resulting in TBI, SCI, strokes, and brain tumours [22] (Figure 2).

The gut and brain connection are inevitable. The gut is completely innervated and controlled by the neurons of the ENS. The imperial connection was initiated during earlier embryogenesis. The initial neural crest later becomes differentiated into the ENS and CNS. In addition, during development, these two systems are connected by the tenth cranial nerve, the vagus nerve, straight from the brain stem to the abdomen [23]. In addition to the GI tract, microbiota also colonizes in the nasal region, as the nasal tract is one of the predominant entry sites of microbes. The nasal mucosa encompasses various microbial communities, which determine olfactory health, and CNS function. Nasal microbiota metabolites can enter the brain through the blood–brain barrier and reach the olfactory epithelium or bulb. Any dysbiosis in nasal microbiota can cause olfactory function intrusion; henceforth, olfactory dysfunction is one of the primary indicators of neurological illness [24].

The human GI tract consists of a collective number of microbial cells. Their metabolites constitute the GM, play a functional role in maintaining physiological and metabolic processes, confer immunity against pathogens [25], and process the brain functions and behaviour of the host [26]. GM produces some microbial products through which it becomes more interactive with the host by entering the circulation from the GI tract to all distant organs [27]. GM responds to diet and exercise and significantly produces changes in the mood and cognition of the host [28]. GM metabolites react with receptors of the brain and synthesize the neuroactive components that affect the GI and mental health of the host. It is described that probiotics can produce neuroactive chemicals and regulate the functions of the CNS as well as the GI tract through neuronal cells and immune cell receptors [29].

Probiotics can work both inside and outside of the GI system. While inside the GI system, the probiotics interact with the gut microbes directly or through their enzymes with the intestinal mucosal layer and epithelial layer, which changes the barrier functions and mucosal immune system. Outside the GI system, probiotics interact with other organs, such as the liver and brain [30]. The ENS and CNS form a complex network that works with the help of neurotransmitters. Any changes in the levels of neurotransmitters can affect CNS function through neuronal signals [10].

## 4. Role of Psychobiotics in Neurological Diseases, Mood States and Neurological Injuries

The brain is fluxed with information from the surrounding environment. The senses collected and received the external stimuli and produced actions to react to the environment [31]. The neuropsychological studies revealed that cognitive functions depend on the brain regions amygdala and the frontal cortex. Mood disorders are characterized by the inability to perform everyday life functions, either professional or social. Persistent depression and mood elevations further influence cognitive functions such as attention and memory [32].

### 4.1. Autism Spectrum Disorder (ASD)

ASD is a highly heritable neurodevelopmental disorder. According to the diagnostic and statistical manual of mental disorders-V (DSM-V) published by the American Psychiatric Association (APA), a person could be autistic only if they displayed 6 of the 12 specified defects in social interaction, communication, and repetitive behaviours, along with the relevant cognitive and motor behaviours [33]. The levels of ASD (mild, moderate, and severe) can be differentiated by the accurate subjects’ examination [33]. The prevalence of ASD is estimated to be under 1% and more in high-income countries. Neuroimaging and electrophysiological studies explained the involvement of anatomical and functional differences in the brain of ASD. The disease management involves psychosocial interventions such as social engagement with language, and communication development, which improves the behaviours of ASD [34].

Mounting evidence stated the link between ASD and gut microbiota. The metabolites synthesized by GM stimulate the production of chemokines, antimicrobial peptides and neuropeptides that coordinates the gut–brain interactions. This bidirectional communication links the gut and the ENS with the cognitive and emotional points of the brain [19]. The neuroactive metabolites from the gut can connect with the distant CNS through the vagus nerves. The catecholamines carry out neurodevelopmental processes such as neurogenesis, microglial activation, brain plasticity, and blood–brain barrier permeability [35]. Autistic children suffer altered gut metabolism and absorption of disaccharides in the gut epithelium and more absorption of mono- and disaccharides into the large intestine, which result in a greater number of fermenting bacteria that lead to changes in GI microbial composition. High sugar concentrations in the large intestine cause gas formation, bloating and osmotic diarrhoea in ASD patients [36].

Probiotic administration can correct gut microbial dysbiosis by modulating the gut microbiota, stabilizing the microbial communities, and restoring the gut environment from the overproduction of harmful metabolites [37]. Intake of probiotics has several health benefits by positively regulating gut microbiota. In addition, probiotic interventions in ASD children can be considered an alternative treatment methodology or therapeutic supplement [38]. Probiotics promote gut integrity and control the gut and CNS inflammatory pathways [39] by producing regulatory T cells, reducing LPS levels and boosting the BDNF. Maintaining BDNF levels in the CNS is critically important to promoting the survival of neurons, making synaptic connections and plasticity, and normal neurological development [35].

Probiotics cause immunomodulation in ASD patients by inhibiting the synthesis of pro-inflammatory cytokines such as interleukin 12 (IL 12), tumour necrosis factor-α (TNF-α), interferon-α (INF-α) and enhance the anti-inflammatory cytokines (IL 10) and transforming growth factor-β (TFGF-β) and thereby regulate the gut inflammation, control the immunological functions as well as improve the behavioural problems in ASD. Hence, probiotic supplementation significantly reduces inflammation, anxiety, stress, and depression and improves positive emotions and behavioural symptoms in ASD [40].

The influence of supplementation of the probiotic mixture (*L. acidophilus, L. casei, L. delbrueckii, B. longum* and *B. bifidum*) and prebiotics in ASD children were studied. The results showed that probiotic supplements improved the GI symptoms and autism treatment evaluation domains such as speech, communication, sociability, sensory awareness and physical behaviour, and health [41].

Pre-schoolers with ASD were supplemented with the probiotic mixture (*S. thermophilus, B. breve, B. longum, L. acidophilus, L. plantarum, L. paracasei, L. delbrueckii* subsp. *bulgaricus*) for 6 months. Then, ASD assessments were carried out using standard ASD scales. Children with GI symptoms showed significant changes in multisensory processes and adaptive functioning [42].

A case study in a 12-year-old ASD child with a severe cognitive disability showed that supplementation of the probiotic mixture (*B. breve, B. longum, B. infantis, L. acidophilus, L. plantarum, L. paracasei, L. bulgaricus, delbrueckii* subsp., *S. thermophilus, S. salivarius* subsp.) for 4 weeks could reduce the autistic symptoms and its severity with increased social affect domain score [43].

**Figure 3:**
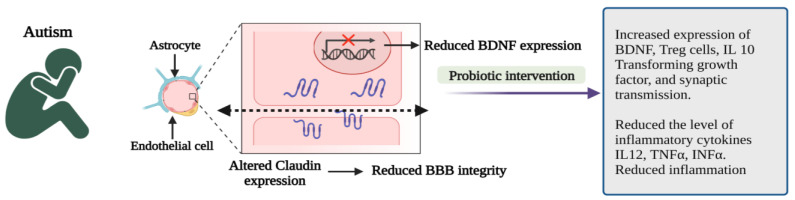
Pathophysiological features and impact of probiotic interventions in autism spectrum disorder (ASD) [35,40]. (Figure created using BioRender.com; accessed on 17 October 2022.)

The oral administration of human commensal *Bifidobacterium fragilis* (*B. fragilis*) enhanced the gut microbiota, gut physiology, permeability and ASD-like behavioural disruptions in the mouse model [44]. Administration of *Lactobacillus reuteri* (*L. reuteri*) restored the neurotransmission and social deficits [42] and diminished stress-induced corticosterone levels in the ASD model [45] (Figure 3).

### 4.2. Parkinson’s Disease (PD)

The DSM-5 framed a common criterion for diagnosing neurocognitive disorders with common cognitive symptoms and defined the aetiological differences. Accordingly, PD is a neurocognitive disorder characterized by genetic factors and a high prevalence of Lewy body deposition [46] in the dorsal motor nucleus of the medulla oblongata and vagus nerves. The Lewy inclusions spread over the CNS, substantia nigra and cortical regions. Another pathological reason behind PD is α-synuclein accumulation in the CNS, which arises due to mutations in the α-synuclein genes. α-synuclein accumulation results in impaired neuronal transport, synaptic plasticity, and neurodegeneration. PD possesses characteristic motor, non-motor, and GI symptoms. This indicates that PD induces alterations in the gut microbiota. The altered gut composition causes changes in lipid metabolism, immunoregulation, and gut permeability, which are the attributes of PD pathogenesis. Brain pathophysiology is linked with gut dysfunctions. Gut dysbiosis affects brain tissue and causes systemic inflammation [47].

The abundance of *Dorea longecatena* (*D. longecatena*), *Bacteroides massiliensis* (*Ba. massiliensis*), *Bacteroides coprocola* (*Ba. coprocola*), *Bacteroides plebeius* (*Ba. plebeius*), *Stoquefichus massiliensis* (*Stoq. massiliensis*), *Prevotella copri* (*Pr. copri*), *Faecalibacterium*, *Blautia glucerasea* (*Bl. glucerasea*), *Ruminococcus callidus* (*R. callidus*), *Coprococcus eutactus* (*Co. eutactus*) and *Bacteroides dorei* (*Ba. dorei*) was decreased, and the abundance of *Catabacter hongkongensis* (*Ca. hongkongensis*), *Lactobacillus mucosae* (*L. mucosae*), *Bifidobacterium*, *Papillibacter cinnamivorans* (*Pa. cinnamivorans*), *Oscillospira*, *Christensenella minuta* (*Ch. minuta*) and *Ruminococcus bromii* (*Ru. bromii*) was increased in the PD patients [48].

Probiotic interventions improve gut microbial composition and health by reducing inflammatory responses, improving the host’s antioxidant system, neuroprotective attributes and neuroinflammation [49]. The supplementation of probiotic tablets containing *L. acidophilus* and *B. infantis* for over 3 months reduced abdominal cramps and bloating in PD patients [50]. The supplementation of fermented milk containing *L. casei* Shirota (6.5 × 10^9^ CFU) for 6 weeks improved stool consistency and bowel habits and reduced bloating and abdominal pain in PD patients [51]. The consumption of the probiotic mixture (*L. rhamnosus* GG, *L. acidophilus, L. plantarum, L. paracasei, L. delbrueckii* subsp. *bulgaricus* and *Bifidobacterium* sp.; 250 × 10^9^ CFU per day for 4 weeks) enhanced the bowel movements and bowel frequency in PD patients with Rome III criteria constipation [52].

Peripheral blood mononuclear cells (PBMCs) isolated from PD patients were cocultured with probiotic strains (*L. salivarius* LS01, *L. plantarum* LP01, *L. acidophilus* LA02, *L. rhamnosus* LR06, *B. breve* BR03 and *B. animalis* subsp. *lactis* BS01) in 1:1 ratio. The PBMCs with *L. salivarius* LS01 and *L. acidophilus* LA02 showed a significant reduction in the studied pro-inflammatory cytokines and increased anti-inflammatory cytokines [53].

Borzabadi et al. reported that probiotic supplementation (*L. acidophilus, B. bifidum, L. reuteri, L. fermentum*) enhanced the expression of IL-1, IL-8, TNF-α, TGF-β, and PPAR-γ, without significantly affecting the expression of vascular endothelial growth factor (VEGF) and low-density lipoprotein receptor (LDLR) in PD patients [54].

6-OHDA-treated C57BL/6 mice and human SH-SY5Y cells were treated with a probiotic formulation containing *Streptococcus thermophilus* DSM 32245 (*Strep. thermophilus* DSM 32245), *B. lactis* DSM 32246, *B. lactis* DSM 32247, *L. acidophilus* DSM 32241, *L. helviticus* DSM 32242, *L. paracasei* DSN 32243, *L. plantarum* DSM 32244, *L. brevis* DSM 27961, and the impacts of the probiotics were observed. Probiotic supplementation increased the expression of neuroprotective protein levels and reduced the proteins responsible for neuronal death. The results showed the protective effect of probiotics in dopaminergic neurons and developments of behavioural attributes, as well as counteract oxidative damage and neuroinflammation via increased BDNF neuroprotective and survival pathways by activating PPAR-γ (peroxisome proliferator-activated receptor-γ), anti-inflammatory and antioxidant activities [55].

The probiotic mixture containing *L. rhamnosus* GG, *L. acidophilus* and *Bifidobacterium animalis* subsp. *lactis* (*B. animalis* subsp. *lactis*) enhances the synthesis of butyrate that helps elevate the levels of BDNF; GDNF (glial cell-line derived neurotrophic factor), which promote the neuronal cell survival, proliferation, dopamine synthesis, and survival of dopaminergic neurons in 1-methyl-4-phenyl-1,2,3,6-tetrahydropyridine (MPTP) induced neurotoxicity condition. *L. rhamnosus* GG reduces the monoamine oxidase B expression in the striatum and increases neurotrophic factors in the brain [56].

The supplementation of *Bacillus subtilis* (*Bac. subtilis*) PXN21 triggered sphingolipid metabolism in the host. It maintained the levels of lipid types such as ceramides and sphingolipids and restored the neuroprotective effects by producing biofilm in the gut of *Caenorhabditis elegans* [57].

Gut non-motor symptoms (GNMS) are prevalent in PD patients. Treating GNMS would be advantageous in alleviating gut dysbiosis and restoring bowel functions. Prolonged use of drugs such as benserazide and dopamine agonists might disturb dopaminergic neuron functions. PD patients were co-treated with Probio-M8 and benserazide, dopamine agonists, for 3 months. Probio-M8 and the drug-co-administered group showed improved sleep and reduced anxiety and GI symptoms. In addition, the metagenomic analysis revealed that probiotic intervention significantly increased the abundance of *B. animalis*, *Ruminococcaceae,* and *Lachnospira* and decreased the abundance of *L. fermentum* and *Klebsiella oxytoca*. Thus, the Probio M8 co-administration enhanced the organisms involved in synthesizing GABA, SCFA, secondary bile acids, serum acetic acid and dopamine. The modification effects of ProbioM8 help manage the gut microbiome, thereby modulating the gut metabolism and managing GNMS symptoms in PD [58].

The results showed that probiotics could ameliorate neurodegeneration and restore brain health in PD subjects. The studies in PD models suggested that regular probiotics supplementation could be a promising strategy for PD treatment, management, and prevention.

### 4.3. Alzheimer’s Disease

National Institute on Aging and the Alzheimer’s Association (NIA-AA) and DSM-5 describe AD as a neurocognitive disorder with the gradual decline or loss of cognitive abilities, with potential biomarkers such as accumulation of amyloid beta Aβ and phosphorylated tau protein, some AD cases predisposed due to genetic mutation [59]. The accumulation of hyperphosphorylated *tau* protein and neurofibrillary tangles in AD patient’s brain led to cognitive and memory impairment [60]. Aβ deposition and *tau* hyperphosphorylation are major traits of AD pathogenesis. Aβ accumulates in the form of neurofibrillary tangles in the neuronal cells, leading to *tau* pathology and extensive loss of neurons, mitochondrial malfunctioning, and neurodegeneration. Aβ accumulation also decreases axonal transport and hinders synaptic transmission [60]. Aβ peptides occur due to the proteolytic cleavage of the amyloid precursor protein, and brain-specific *tau* proteins become hyperphosphorylated and lose their affinity with microtubules of axons, thus hindering the cognitive processes and causing neuroinflammation, neuronal loss and neurotransmitter imbalance and synaptic loss [61].

The progression of AD is associated with aging, poor diet, and inflammatory responses in the gut. GI dysfunction due to aging could affect CNS and cause neurological complications. The gut microbial environment can be positively modulated with the help of probiotics and a probiotic-rich diet [62]. The abundance of organisms belonging to the genera *Verrucomicrobia, Firmicutes, Proteobacteria, Bifidobacterium* and *Actinobacteria* was drastically reduced, and members of genera *Tenericutes*, *Bacteroidetes* were raised in AD conditions [63]. Intake of probiotics could reconstitute the colonization of gut microbes and can modulate AD progression by improving immune responses [64]. Probiotic supplementation enhances the serum tryptophan metabolism in AD patients [9].

AD patients aged 60–95 years were supplemented with 200 mL of probiotic milk containing *L. acidophilus, L. fermentum, L. casei* and *B. bifidum* per day for 12 weeks. The changes in their cognition were accessed by Mini-Mental State Examination (MMSE), and biochemical markers such as inflammation, oxidative stress and metabolic profiles were also measured. The MMSE cognitive score in probiotic-treated AD subjects was +27.90% ± 8.07, significantly higher than in the control (−5.03% ± 3.00). AD patients are prone to oxidative stress, dyslipidaemia, and insulin resistance. Probiotic-supplemented AD subjects showed favourable levels of malondialdehyde (MDA), serum high-sensitivity C-reactive protein (hs-CRP), triglyceride and markers of insulin metabolism. Still, no significant changes were observed in oxidative stress and inflammation markers. The MMSE results imply that probiotic treatment could reverse cognitive decline and improve cognitive symptoms [65].

Similarly, another randomized, controlled clinical trial study was conducted among seventy-nine AD patients aged 55–100. They were supplemented with probiotic *L. acidophilus*, *B. longum*, *B. bifidum*, and selenium for 12 weeks. The probiotic plus selenium supplemented group showed improved MMSE cognitive score and favourable effects on insulin metabolism markers and hs-CRP, very low-density lipoprotein (VLDL), low-density lipoprotein (LDL), and total high-density lipoprotein (HDL) cholesterol levels. The probiotic and selenium co-supplementation group showed increased expression of PPAR-γ and LDLR and reduced TNF-α expression. The probiotic supplementation did not affect the inflammation, oxidative stress markers, or lipid profiles [66]. The probiotics with selenium could attenuate oxidative stress and inflammation with the help of metal ion chelating activity and antioxidant system through regulating NFκB and MAPK pathways [67,68].

The effects of the probiotic mixture (*Strep. thermophilus*, *B. longum*, *B. breve*, *B. infantis*, *L. acidophilus*, *L*. *plantarum*, *L. paracasei*, *L. delbrueckii* subsp. *bulgaricus*, *L. brevis*) in the AD mouse model has been reported. Its impact on cognitive decline was measured using novel object recognition and passive avoidance tests with the help of probes in the mouse’s hippocampus and amygdala regions. Probiotic supplementation restored hippocampal functions with improved behavioural performance in AD mice. Probiotic administration enhanced energy, amino acid, and nucleotide metabolic pathways. The abundance of *Bifidobacterium* strains was increased, and *Campylobacter* strains were decreased in AD mice. The circulating pro-inflammatory cytokines, IL-1α, IL-1β, IL-2, IL-12, interferon-γ (INF- γ), and TNF- α levels are reduced upon probiotic treatment compared to control AD mice, indicating the inflammatory regulatory effects of the studied probiotics. Probiotics treatment significantly reduced AD mice’s Aβ load at 12, 18 and 24 weeks. Probiotic intervention induces changes in microbial communities in AD mice and exhibits modification of microbiota-dependent anti-inflammatory effects in AD models. The inhibition of pro-inflammatory cytokine signals reduces Aβ deposition and restores cognitive impairment in the AD mouse model [60].

Probiotic supplementation (*L. acidophilus*, *B. bifidum* and *B. longum*; 15 × 10^9^ CFU for 6 weeks) positively regulates long-term potentiation by increasing presynaptic neurotransmitters’ release, improving the spatial learning and memory in AD-induced rats [69]. Oral administration of probiotics (*L. reuteri, L. rhamnosus* and *B. infantis*; 10^10^ CFU for 10 weeks) significantly improved spatial memory and reduced Aβ plaques in the brain of AD rats. In addition, IL-1β and TNF-α levels were decreased, and the excitatory postsynaptic potential increased in the experimental rats’ hippocampus [70]. *B. infantis* administration reduces Aβ deposition, IL-1β, and TNF-α levels and modulates neuroinflammation by retaining the immune responses, reducing cognitive dysfunction. *B. infantis* administration also suppressed Aβ toxicity and supported the expression of BDNF, which helps in neuronal survival [71].

Intragastric treatment of *Clostridium butyricum* (*Cl. butyricum*) to APPswe/PS1dE9 transgenic mice for 4 weeks prevents cognitive impairment and Aβ deposition and activates the microglial cells. *Cl. butyricum* treatment inhibited the production of TNF-α and IL-1β in the transgenic mice brain. The results indicated that *Cl. butyricum* treatment regulates AD progression [72]. Similarly, eight weeks of probiotic supplementation (*L. acidophilus, L. fermentum, B. lactis* and *B. longum*; 1 × 10^10^ CFU) significantly improved the spatial learning and memory in Aβ1-42 infused AD rats and inhibited AD pathology by regulating the GM [73].

Kobayashi and team examined the effect of *B. breve* A1 in ameliorating cognitive impairment in AD mice. The daily administration of *B. breve* A1 reduces memory dysfunction. It suppresses Aβ-induced changes in the hippocampal gene expressions, which is responsible for inflammation and immune reactive genes. It significantly enhances the acetate levels but does not affect other short-chain fatty acids (SCFA) and the gut microbiota. The probiotic treatment ameliorates the behavioural deficits induced by Aβ in the mice model [74].

**Figure 4:**
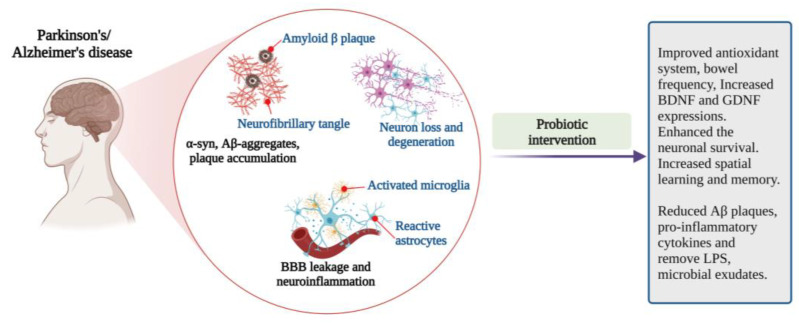
Pathophysiological features and impact of probiotic interventions in Parkinson’s and Alzheimer’s diseases [46,51,53,54,55,56,57,58,70,71,74] (Figure created using BioRender.com; accessed on 17 October 2022).

The studies claimed that probiotic supplementation positively correlates with the changes in gut microbial composition and decelerates the disease progress. However, large-scale clinical trials with human subjects are required to establish an effective probiotics-based therapeutic formulation for AD (Figure 4).

### 4.4. Mood States: Anxiety, Stress, Depression

**Figure 5:**
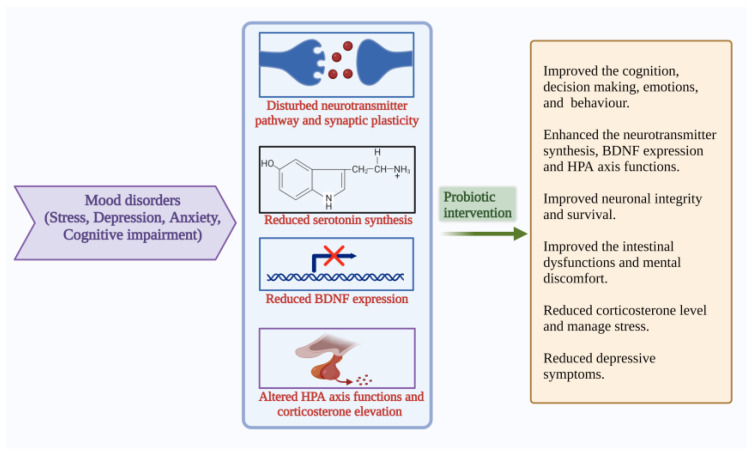
Pathophysiological features and results of probiotic interventions in mood disorders such as stress, depression, anxiety, and cognitive impairment [9,10,13,14,42,45]. (Figure created using BioRender.com; accessed on 17 October 2022.)

APA described that mood, anxiety and eating disorders are Axis I disorders. Anxiety is associated with more than three symptoms, including restlessness, fatigue, irritability, muscle tension and disturbed sleep, which cause stress [75]. Extreme mood swings, loss of energy, sadness, sleep impairment, lack of concentration, attention, difficulty in decision-making, anhedonia and heterogeneity of symptoms are the characteristics of depression [76]. The gut microbiome can regulate the metabolism, which further influences gut–brain communication and is associated with GI and psychiatric illness. The commensals control nerve functions through their metabolites and are combined with several pathways [77]. The gut and brain crosstalk has gained more scientific attraction due to their complex communication strategies. The gut–brain bidirectional communication becomes more important for GI and psychiatric health. [77]. GI homeostasis becomes disturbed in mood disorders and may result in cognitive decline. Maintaining gut microbial balance is important to learn psychology and cognitive functions. Anxiety, depression, and stress, together with comorbid conditions are highly influenced by each other and intertwined. Mental health is related to dietary habits. Several studies explain the role of probiotic-based food supplementation in the cognitive function of humans. These studies recommend that probiotic consumption might help improve cognition, decision-making and stress management [78] (Figure 5).

The first way of connection between the gut and brain is through immunoregulation. Gut microbes interact with immune cells of the lymphatic system and affect inflammation, immune response, and cytokine production [79]. The second connection is through the vagus nerves, which connect the entire GI region with the brain [80]. Neurons exposed inside the gut regions interact with gut microbes that regulate the secretion of gut hormones. The microbial metabolites interact with vagus nerves and connect to the brain, regulating various brain functions, including hormone release, sleep, and stress response [81]. The third way of interaction is through the neuroendocrine system. Gut microbes modulate neurotransmitter synthesis and availability [79]. In the case of anxiety and depression, the GM influences the tryptophan–kynurenine pathway [82], which particularly resists the conversion of tryptophan into serotonin, leading to serotonin depletion and the development of anxiety and depression [83].

**Figure 6:**
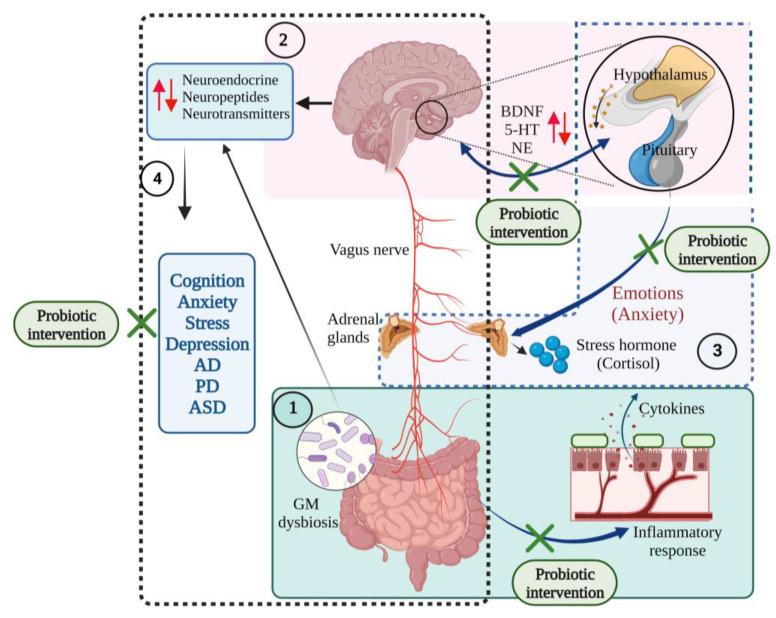
The communications of microbiota–gut–brain–hypothalamus–pituitary adrenal connections in neurological and mood states. (1) Gut dysbiosis initiates inflammatory responses, producing proinflammatory cytokines that affect the central nervous system and cause gut disorders. (2) Brain–hypothalamus–pituitary axis regulates the expression of brain-derived neurotrophic factor (BDNF), serotonin (5-HT), and norepinephrine (NE) levels and maintains mood states (several emotions such as anxiety). (3) Hypothalamus–pituitary–adrenal axis regulates the synthesis of the stress hormones such as cortisol and responds to mood states. (4) Gut microbiota-brain axis controls the synthesis of neuroendocrine, neuropeptides and neurotransmitter molecules. Gut microbial (GM) dysbiosis induces changes in the production of these neuronal molecules and results in neurodegenerative diseases and mood disorders. Probiotic interventions restore the gut eubiosis and improve its functions, improve the immune functions, and regulate the gut–brain interrelationship [10,11,19,46,77,91,92]. (Figure created using BioRender.com; accessed on 18 October 2022.)

The gut–brain axis modulates the HPA axis, which controls mood, emotion, and BDNF expression [84]. Hippocampal expression of BDNF and serotonin receptor 5HT1A are frequently related to emotion and anxiety-like behaviour [79,85] Serotonin receptors receive input from neurons of the dorsal and ventral raphe nucleus plays an important role in anxiety-like behaviours [86]. In addition, the N-methyl-D-aspartate (NMDA) receptor subunits NR1, NR2A, and NR2B were also found to play an important function in synaptic plasticity, learning and memory, as well as anxiety [87]. Agonists of the 5HT1A receptor produce the anxiogenic effect, and any changes in the 5HT1A receptor would alter the anxiety behaviour, evidence that serotonergic signalling contributes to the anxiety-like behaviour [88]. *B. infantis* intervention significantly reduced the hyper response of the HPA axis and 5-HT and non-epinephrine (NE) levels in the hippocampus and cortex of germ-free mice [77,89]. Probiotic treatment with *B. infantis* intervention reversed the behavioural deficits in the forced swim test, reversed the basal NE level in the brainstem, and normalized the immune responses in the rat maternal separation model of depression [89]. Probiotic supplementation (*B. longum* 1714 and *B. breve* 1205 for 6 weeks) reduced anxiety and depressive behaviour in the mice model [90] (Figure 6).

Distress reduces the hippocampal BDNF expression, activates the HPA axis and increases anxiety. The reduced expression of BDNF in the hippocampal dentate region causes anxiety-like behaviour [83,93,94]. The high proportion of stress, depression and anxiety is linked with intestinal dysfunctions, gut disorders, and mood disorders [95,96] due to altered gut and CNS functions and interactions [91]. Thus, the beneficial effects of probiotics to improve stress and cognition are linked with the HPA axis.

The supplementation of fermented milk containing Lactobacillus delbrueckii subsp. bulgaricus (*L. delbrueckii* subsp. *bulgaricus*), *L. casei* and *Streptococcus salivarius* subsp. *thermophilus* (*Strep. salivarius* subsp. *thermophilus*) for 6 weeks reduced the risk of infection by regulating the immune players. They maintained the cortisol level in students under academic examination stress [92]. The supplementation of *L. plantarum* P-8 for 12 weeks significantly reduced the stress and anxiety in stressed adults. In addition to anxiolytic effects, positive changes in GM were also observed. The production of neurotransmitters and neuroactive metabolites, including SCFA, GABA, and arachidonic acid, was also enhanced [97].

The effect of *B. longum* NCC3001 supplementation on anxiety and depression in irritable bowel syndrome patients with psychiatric illnesses was studied. The results showed that *B. longum* reduced depression and increased the quality of life of the subjects with no significant changes in their anxiety scores, attributed to the changes in brain activation patterns by reducing limbic reactivity [98]. 

MDD patients aged 20–55 years were supplemented with probiotic capsules containing *L. acidophilus*, *L. casei* and *B. bifidum* for 8 weeks. After the supplementation, they showed a decrease in Beck Depression Index scores (BDIS) and mood improvement [99]. It is likely that the supplementation of *L. helviticus* and *B. longum* for 8 weeks improved the BDIS, increased the tryptophan/isoleucine ratio, and decreased the kynurenine/tryptophan ratio significantly compared to placebo in the probiotic group in MDD patients [100].

The supplementation of (*B. bifidum* W23, *B. lactis* W52, *L. acidophilus* W37, *L. brevis* W63, *L. casei* W56, *L. salivarius* W24 and *L. lactis* W19 and W58) for 4 weeks reduced cognitive reactions to sad mood states, assessed through Leiden index of depression sensitivity scale, in young, healthy subjects [101]. Similarly, *L. helviticus* R0052 and *B. longum* R0175 supplementation for 30 days reduced the hospital anxiety and depression scores (HADS) and cortisol levels. It enhanced the emotional responses in healthy volunteers [11]. The effect of fermented milk supplementation, which contains *B. animalis lactis, Streptococcus thermophilus, L. bulgaricus* and *L. lactis* over 4 weeks was reported on healthy female subjects’ brain activities. The study subjects were allowed to investigate negative emotions such as fear and anger; meanwhile, their brain activities were recorded by functional magnetic resonance imaging. The brain regions responsible for emotion processing, including the insula, somatosensory cortex, and periaqueductal grey, showed reduced activities in the probiotic group. The result indicated that probiotics could modulate emotional activities in healthy individuals [102]. A meta-analysis was carried out to evaluate the role of probiotics in MDD. The study results indicated that combinational probiotic (with different strains of probiotics) treatment reduced depressive symptoms and improved the gut–brain axis in MDD subjects [103].

### 4.5. Cognitive Impairment

Cognition is a constructive health marker [104] used to measure psychological health, especially in adolescents [105]. Cognitive dysfunction is a common symptom of several psychiatric disorders. Dementia and MCI are two varied entities of cognitive impairment. When cognitive impairment is found to compromise social functioning and memory impairment, it might be a symptom of dementia. MCI is an intermediate stage between normal cognition and dementia with functional disabilities [106]. According to the DSM-V, substantial impairment in one or more cognitive domains (acquisition, retention, and usage of knowledge) might cause neurocognitive disorder and interfere with day-to-day activities [76].

Cognitive dysfunction is a complex disorder accompanied by disturbances in various factors such as attention, learning, psychomotor speed, computation, problem-solving, decision making and executive functioning [107]. Poor cognitive skills during adolescence are associated with a higher risk of anxiety disorders, depression, stress, coronary heart disease, and cancer in later life [108]. Cognitive performance and development relate to the psycho-physiological cerebral functions [109]. Behavioural and neuroimaging studies exposed that any disturbances or impairments in cognition may cause mental illnesses, major mood disorders, bipolar disorders, and schizophrenia. The potential markers or biological causes for cognitive deficits need to be elucidated to treat serious mental illnesses [110]. Cognitive decline due to stress conditions can be reversed using probiotics. Probiotics positively regulate the gut epithelial barrier, reduce intestinal permeability and boost memory performance [111]. Henceforth, the substantial impairment in one or more than one cognitive domain might result in neurocognitive disorder and interfere with day-to-day life activities.

The function of multiple-species probiotics on neurocognition and stress has been reported. Probiotic [Ecologic^®^ barrier; *Bifidobacterium bifidum* (*B. bifidum*) W23, *Bifidobacterium lactis* (*B. lactis*) W52, *Lactobacillus acidophilus* (*L. acidophilus*) W37, *Lactobacillus brevis* (*L. brevis*) W63, *Lactobacillus casei* (*L. casei*) W56, *Lactobacillus salivarius* (*L. salivarius*) W24, *Lactobacillus lactis* (*L. lactis*) W16 and *L. lactis* W58] supplementation (5×10^9^ CFU for 4 weeks) causes differences in cognition under an induced stressed condition in healthy human subjects. It shows that probiotics supplementation triggers stress-related increases in working memory performance and brain functions during stress conditions, which was associated with intervention-related neural changes in the frontal cortex [112].

Cognitive impairment commonly prevails in the elderly and is associated with aging, dementia, and AD-like disorders. The effect of supplementation of *Bifidobacterium breve* A1 (*B. breve* A1) on cognitive function was studied in elders with mild cognitive impairment (MCI). In detail, the elderly subjects were supplemented with *B. breve* A1 for 24 weeks. Their cognitive abilities were evaluated using a mini-mental state examination (MMSE) and digit symbol substitution test (DSST). The subjects’ mental condition and GI symptoms were studied by Profile of mood states 2nd edition (POMS2) and the GI symptom rating scale (GSRS). The results showed that oral probiotic supplementation improved cognitive function and decreased the risk of dementia in MCI patients [113].

Similarly, elder subjects (50–80 years old) with subjective memory complaints were supplemented with *B. breve* A1 for 12 weeks. The cognitive standards, including immediate memory, visuospatial, language, attention, and delayed memory, were analyzed using Repeated Battery for the Assessment of Neurophysiological Status (RBANS), and cognitive functions were evaluated with the help of MMSE. RBANS and MMSE scores were significantly increased above baseline after probiotic treatment. The results indicated that *B. breve* A1 supplementation could provide beneficial effects against cognitive decline or memory impairment in the elderly [114].

Preclinical studies in healthy volunteers showed that consuming psychobiotics affects stress, cognitive response, and brain activity patterns. The consumption of *Bifidobacterium longum* (*B. longum*) 1714 improves stress-related behaviours and cognitive performances. Cognitive performances were accessed by evaluating attention, memory, social and emotional cognition, and processing. Brain patterns were analysed using electroencephalogram (EEG) in the brain’s frontal, parietal and central regions of volunteers who consumed *B. longum* 1714 for 4 weeks. Improved visuospatial memory performance and memory in EEG profile revealed that the strain *B. longum* 1714 could act as a putative psychobiotic by exhibiting anti-stress and precognitive effects and alleviating the acute stressor responses [17]. Several studies showed that *Bifidobacterium breve* (*B. breve*)*, Bifidobacterium infantis* (*B. infantis*), *B. bifidum*, and *L. acidophilus, Lactobacillus helviticus* (*L. helviticus*), *L. rhamnosus, Lactobacillus plantarum* (*L. plantarum*), *Lactobacillus sporogenes* (*L. sporogenes*), *Lactobacillus bulgaricus* (*L. bulgaricus*), *Lactobacillus delbrueckii* (*L. delbrueckii*), *L. casei, L. salivarius* and *Lactobacillus paracasei* (*L. paracasei*) produce a significant impact in the neurotransmission and CNS functions and emotional states related to mental disorders [44,115,116,117,118].

Another clinical study evaluated the impact of *L. plantarum* C29-fermented soybean (DW2009) supplementation for 12 weeks in healthy subjects with MCI. Cognitive scores were measured using computerized neurocognitive function tests, and the BDNF levels in serum changes were examined. DW2009 supplementation improved attention and cognition, attenuated memory impairment and increased serum BDNF expression. Increased BDNF levels after the DW2009 administration showed the involvement of probiotics in the survival of neurons and cognitive improvement [119].

The studies support that probiotic supplementation improves cognitive development and neuronal health. Probiotics directly impact the host’s intestinal microbiota and are not harmed by host enzymes, stimulate the growth and activities of beneficial gut microbiota, and provide positive effects on cognition and other mood states by modulating the gut–brain axis signals [120] (Figure 4).

Probiotics could alleviate depressive symptoms in patients with depression. Still, the role of probiotics in managing changes in mood states is unclear; thus, several studies require exploring the molecular mechanism behind the effects of the probiotic intervention. Regularly consuming probiotics and fermented food containing probiotics might be prudent for managing disturbing mood states (Table 1).

### 4.6. CNS Injuries and Probiotics

The bidirectional communication between the brain and GI tract is important for maintaining homeostasis. The neuronal innervations inside the gut and the enteric nerves regulate the blood flow, hormone release and peristalsis; on the contrary, the gut microbes regulate the synthesis of SCFAs, neurotransmitters, mood, inflammation, and injuries [18]. Neuroinflammation and elevated oxidative stress in the neuronal environment may induce apoptosis and neurodegeneration [131]. Even though TBI occurs due to brain damage by external forces, the TBI-related pathophysiological events cause changes in the BBB, oxidative stress, and immune response to neuroinflammation, astroglia activation and mitochondrial dysfunction [132]. 

Recent studies suggested that probiotics could be an adjuvant therapy treatment for TBI management. The human preclinical trial studies revealed that TBI-injury patients supplemented with Lactobacilli-rich probiotics within 48 h of hospitalization would noticeably reduce GI dysregulation [133]. Other CNS injuries such as ischemic stroke, SCI and hemorrhagic cerebrovascular lesions are linked with altered GM composition, modulated SCFAs concentrations, hormone and neurotransmitter release and inflammation [18]. The probiotics, which could produce SCFAs such as butyrate, propionate, and acetate, may help maintain mitochondrial functions and homeostasis in TBI and SCI injuries [134]. The butyrate derived from Clostridium butyricum improved neurological deficits, and attenuated neurodegeneration reduced brain edema and BBB integrity in cerebral ischemia [135]. Thus, the probiotics might diminish the infections in brain injuries and help balance the GM.

## 5. Diet and Neuroprotection 

Functional foods with their nutritional values provide several health benefits, including cognitive development and mental health. The presence of probiotic strains and the bioactivity of nutrition are some of the important reasons for the health-enhancing effects of functional foods [136]. 

The brain requires energy for its function and to regulate the whole body. The disturbance in energy transfer could alter synaptic plasticity, metabolic activities, and cognitive processes [137]. Neurological diseases have their connection with the gut. The interaction of the gut and brain is partially mediated through GM residing in the GI. Gut microbes are essential for balancing human health, and their changes modulate or regulate the occurrences of diseases including metabolic disorders, GI diseases, neurological diseases, and cancers [138]. The gut microbes influence the development and functions of CNS and ENS and are regulated by each other. The CNS regulates the ENS through the HPA axis, sympathetic and para-sympathetic nerves [26], neuroendocrine, endocrine pathways, vagus afferents and receptors in the brain [139]. 

The growth and maintenance of gut microbes depend on various factors, including diet, environment, age, and medications [140]. Most prominently, dietary practices directly affect GM [141]. Diet rich in high fat, low fibre, and high sugar can influence the gut microbiota, indicating that diet is one of the strong external factors correlating to the host’s microbiota [142]. Diet could influence synaptic plasticity, brain health and mental functions. In concordance with diet, several gut hormones enter the brain, influencing cognitive ability. The brain regulators such as BDNF respond to food intake [143]. Neurotransmitter pathways, synaptic transmission, cognitive abilities, membrane fluidity and signal transduction pathways are modulated by dietary nutrients. Calories in the diet influence cognitive ability. Excess calories can inhibit synaptic functions and cause neuronal cell damage. A moderate caloric intake can help protect the brain cells from oxidative damage [144].

A high amount of saturated fat diet disturbs the cognitive process and increases the risk of neurological dysfunctions in humans and animals [145,146]. The brain regions that control eating behaviour and cognition are integrated. The consumption of any undesirable food produces aversiveness towards its flavour. Then, the hippocampus, amygdala and hypothalamus develop a memory of that food through learning and memory systems [143]. The reduced consumption of omega-3 fatty acids might be the reason for the increased incidence of depression and other mental disorders [143]. A decrease in brain docosahexaenoic acid (DHA) causes cognitive decline and the on-set of sporadic AD. DHA deficiency is also associated with other psychological illnesses such as attention deficit hyperactive disorder (ADHD), depression, aggressive hostility, cognitive disability and AD, impaired learning and memory [147], dyslexia, dementia, and schizophrenia [143,148,149]. 

Fish is an important source of n-3 polyunsaturated fatty acids, including DHA and eicosapentaenoic acid (EPA). DHA and EPA have an important role in brain function, neurocognitive development, anti-inflammation and maintaining blood pressure [150]. Omega-3 fatty acids-rich diet strengthens the cognitive functions in humans [151] and rodents by positively regulating genes responsible for synaptic functions [152]. DHA is found abundant in the brain cells, which cannot be synthesized by humans efficiently, and is largely derived from the diet; that plays an essential function in the growth and development of the brain, especially in infants, and is necessary for normal brain functions, promotes learning ability [137]. 

In addition to fish products, nuts, especially walnuts, possess a high concentration of monounsaturated fatty acids (MUFAs), polyunsaturated fatty acids (PUFAs), and antioxidant and anti-inflammatory components. Regular intake of nuts positively affects the cognitive performances of young adults and older people [153] and prevents mild cognitive impairment in AD [154].

Linolenic acid, arachidonic acid, α-linolenic acid, EPA, and DHA are classified under PUFAs, highly involved in the preventive functions against dementia and maintaining cognitive functions. Especially DHA in the phospholipid membrane of neuronal cells supports neuronal integrity and health during brain aging. Thus, DHA is potentially involved in regulating genes necessary for cognitive functions, neurogenesis, and neuronal functions [155]. On the other hand, MUFAs improve verbal fluency and episodic memory and reduce the occurrence of MCI [156].

Plant-based diets are rich in polyphenols such as flavonoids, lignans, coumarins and tannins. Among these, flavonoids are the most studied polyphenols regarding brain health. Flavonoids produce positive effects against cognitive decline in AD [157]. Flavonoids showed antioxidant effects, reduced learning and memory impairment in cerebral ischemic rodents [158], and increased hippocampal-dependent memory and synaptic densities in mice [159].

Folate is essential for neuronal health, and its deficiency cause depression and cognitive impairment [142]. Folate interacted with other vitamins, B6 and B12, and showed antioxidant and anti-inflammatory activities by reducing IL-1β, TNF-α and CD-40 in macrophages [160]. Deficiency in vitamin B12 increases the IL-6 in blood mononuclear cells of AD patients leading to an increase in inflammatory functions [161]. Vitamin E, abundant in nuts, vegetable oils, green leaves, and cereals, enhances mitochondrial and neurological functions [162]. Vitamin E deficiency promotes CNS oxidative damage in Purkinje neurons of the cerebellum. Vitamin E supplementation reduces oxidative damage, enhances neuroprotective effects, neuronal survival, and recovery [163], and improves neurotransmission in PD models [164].

The polyphenols from green tea possess numerous health benefits such as anti-inflammatory, antioxidant and neuroprotective activities by scavenging the free radicals and reducing inflammatory cytokines and neuronal damage [165]. Grape seed polyphenols inhibit the Tau peptides accumulation and help prevent and treat AD and PD. Wang and team proved that dietary supplementation of grape seed polyphenols could reduce the severity and development of AD and regulate cognitive performance in the AD mouse model [166]. The metabolites produced due to the transformation of polyphenols by gut microbes interfere with the Aβ aggregation in rats [167].

Antioxidant-rich diets produce positive effects on neuronal functions [143]. Micronutrients such as alpha lipoic acid present in meats and vegetables are potent coenzymes involved in the energy homeostasis of mitochondria [168] and help improve memory and inhibit cognitive decline in AD patients [169].

In addition to genetic and demographic factors, nutritional deficits can also be one of the reasons for the cognitive decline during aging [170]. Malnutrition is quite a common factor of cognitive impairment in the aging population. In addition, the older and oldest adults are susceptible to neuronal damage, and their physiological changes could further accelerate neuronal damage [171]. Especially centenarians have a high prevalence of cognitive decline than older and oldest adults [172]. 

Collectively, this review explains that consuming a healthy diet and balanced nutrition could prevent cognitive decline. Adequate nutrients are essential for every individual to maintain metabolic homeostasis and avoid dysfunction, promote immune health, and maintain the integrity of the brain; thus, dietary patterns are inter-linked with physical and mental health. Disease management and therapies for neurological disorders also recommend dietary supplements as adjuvant therapy in addition to pharmaceutical interventions. Understanding the pathophysiology status of every disorder strengthens the development of new approaches or therapies for treating cognitive impairments, neuronal damage, or neuronal death due to neurological diseases.

## 6. Conclusions

Lifestyle, including work, diet, and less or no exercise, is associated with the risk of many physical and mental health problems. Dietary patterns strongly influence the intestinal and CNS through dietary compounds such as vitamins, fatty acids, etc. The gut and CNS connections are well mediated by microbial networks. The gut microbes are necessary for human health and balanced gut and brain functions. Maintaining a healthy GM with the help of probiotics could support optimum MGBA functions. Studies showed that the coordination of GM and the brain is necessary to manage CNS diseases. The interaction between GM and neuro-physiological disorders has been studied in recent decades. Neurological injuries cause gut dysbiosis and affect the BBB, oxidative stress, inflammatory process, and neuronal damage.

Defined pharmacological and adjuvant therapeutic strategies are needed to manage the emergence of neurological diseases due to lifestyle changes. The consumption of probiotics and probiotics-containing foods might retain the gut microbiome and enhance the synthesis of potential metabolites, inducing changes in GI, neurochemical, neuroendocrine and neuroimmune systems through gut microbes. Thus, probiotics could be considered biotherapeutic agents to treat neurological disorders.

The involvement of microbiota in influencing human health and disease need to be elucidated in a more prudent way. In addition, the exact metabolic or functional aspects of probiotics still need to be comprehensively studied. The presence of diverse microbial species is a key controller of human health. Considering the personalized host response towards any probiotics or functional foods would bolster the understanding of their specific relationship. Further, the development of an advanced range of therapeutics for neurological disorders could be enhanced through proper support from the personalized microbiome analysis, and respective remedies would be developed. However, personalized microbiome analysis is expensive and requires technical support from several interdisciplinary sciences, and many questions in the knowledge gaps are not yet answered. More collaborative studies with varied functional therapeutic approaches might prominently open the gate to pursuing and attaining the respective goals and answers for the enigmatic questions.

The detailed literature survey recommends that combining psychotropic drugs, psychobiotics, and a balanced diet could offer synergistic effects, aiding in managing neuronal diseases such as AD, PD, ASD, and other mood disorders. However, the scientific evidence is insufficient to claim that a particular probiotic strain, or a combination of strains, is a potent candidate to treat neurological diseases and disorders. Thus, the current study endorses the need for in-depth clinical studies on the efficiency and welfare of probiotic combinations and dietary supplements against neurological diseases.

## Figures and Tables

**Table 1:** The influence of probiotic interventions on various neurological disorders and mood disorders.

Probiotic Strain	Study Model	Age	No. of Subjects	Dose and Duration	Results	Ref.
Cognition						
Probiotic sticks (*B. longum* 1714)	Healthy volunteers	25.5 mean years	22	The probiotic stick contained maltodextrin, magnesium stearate and 1 × 10^9^ CFU of probiotics per stick; 1 stick per day for 4 weeks	Improved cognitive performance. Improved visuospatial memory performance and anti-stress, precognitive effects	[17]
Ecologic^®^ Barrier (*B. bifidum* W23, *B. lactis* W52, *L. acidophilus* W37, *L. brevis* W63, *L. casei* W56, *L. salivarius* W24, *L. lactis* W16, and *L. lactis* W58).	Healthy female volunteers	18 to 40 years	61	5 × 10^9^ CFU per day for 4 weeks.	Improved cognitive response and working memory performance	[112]
*B. breve A1*	Patients with MCI	-	27	2.0 × 10^10^ CFU per day for 24 weeks	Improved cognitive function	[113]
*B. breve A1*	Older adults with memory complaints	50 to 80 years	121	2.0 × 10^10^ CFU per day for 12 weeks	Improved cognitive function	[114]
*B. bifidum*, *B. infantis, L. helveticus,* fructooligosaccharides and maltodextrin.	C57BL/6J mice	21 days *	-	*Bifidobacterium* (1.9 × 10^8^ CFU/g); *Lactobacillus* (6.4 × 10^9^ CFU/g) were administered with drinking water to female mice from embryonic day 0.5 to postnatal day 21.	Decreased the incidence of ASD Prevented a series of MIA-induced ASD-relevant deficits. Reduced impairments in social behaviour, repetitive behaviour, stereotyped behaviour, depression, and anxiety-like behaviour. Prevented the decrease in PV^+^ neurons and GABA levels	[117]
Protexin^R^ restore (*L. casei*, *L. rhamnosus*, *S. thermophilus*, *B. breve*, *L. acidophilus*, *B. infantis, L. bulgaricus*)	Young male golden Syrian hamsters	-	50	2 × 10^8^ CFU/Kg body weight dissolved in PBS; once a day for 27 days	Ameliorated the glutamate excitotoxicity	[118]
*L. plantarum* C29 mediated fermented soybean (DW2009)	Physically healthy men and women with MCI	55 to 85 years	100	1.25 × 10^10^ CFU/g per day for 12 weeks	Enhanced cognitive function. Increased BDNF levels Attenuated the memory impairment.	[119]
*L. plantarum* (LAB1, LAB11, LAB12), *L. fermentum* (LAB9, LAB10), and *L. casei* (LABPC)	Male ICR mice and BV2 microglia cell lines	2 months	30	*L. fermentum* (10^9^ CFU) and *L. casei* (10^9^ CFU) per day for 28 days	Attenuated the LPS-induced memory deficit. Increased the antioxidants SOD, GSH, and GPx. Reduced the neuroinflammation by decreasing MDA, AChE, and pro-inflammatory cytokines	[121]
Autism spectrum disorder						
Delpro^®^ (*L. acidophilus, L. casei, L. delbrueckii, B. longum* and *B. bifidum*) with lyophilized peptidoglycan, muramyl peptides and DNA motifs derived from *L. rhamnosus* V strain	Children with ASD and frequent GI distress	3 to 16 years	33	2 billion CFU of each strain/ thrice a day for 21 days	Improved GI symptoms and autism treatment evaluation domains such as speech, communication, sociability, sensory awareness, physical behaviour, and health	[41]
DSF Vivomixx^®^ (*S. thermophilus*, *B. breve, B. longum, B. infantis, L. acidophilus,* *L. plantarum, L. paracasei*, *L. delbrueckii* subsp. *bulgaricus*)	ASD children	18 to 72 months	85	450 billion/ packet. Two packets/day in the first month; 1 packet/day in the next 5 months	Showed positive effects on core autism symptoms. Significantly improved GI symptoms, multisensory processing, and adaptive functioning.	[42]
VSL#3 (*L. delbrueckii* subsp. *Bulgaricus*, *L. acidophilus, B. breve, B. longum, B. infantis*, *L. paracasei, L. plantarum, S. thermophiles*)	ASD children	12 years	-	9 × 10^10^ CFU/g of *B. breve, B. longum, B. infantis*; 8 × 10^10^ CFU/g of *L. acidophilus, L. plantarum, L. paracasei, L. bulgaricus, L. delbrueckii* subsp.; 20 × 10^10^ CFU/g of *S. thermophilus* and *S. salivaricus*/ day for 4 weeks	Reduced the severity of abdominal symptoms. Autistic core symptoms improved. The score of the social affect domain improved	[43]
*Lactobacillus plantarum* WCSF1	ASD children	4 to 16 years	-	4.5 × 10^10^ CFU per day for 12 weeks	Increased the abundance of Enterococci and Lactobacilli. Decreased the abundance of Clostridium cluster XIVa. Improved stool consistency and behavioural scores	[122]
A mixture of 3 *Lactobacillus* strains, 2 *Bifidobacterium* strains, and one *Streptococcus* strain (60: 25: 15 ratio)	ASD children and their non-autistic siblings and other healthy children as control	ASD (2 to 9 years) Siblings (5 to 7 years) Control (2 to 11 years)	ASD (*n* = 10); Siblings (*n* = 9); Control (*n* = 10)	4 months	Probiotic supplementation normalized Bacteroidetes/Firmicutes ratio. Decreased the abundance of *Desulfovibrio* spp. and decreased TNFα level in feces	[123]
*L. plantarum* PS128	ASD boys	7 to 15 years	80	3 × 10^10^ CFU per day for 4 weeks	Ameliorated defiance behaviours. Improved the Swanson, Nolan, Pelham-IV-Taiwan version (SNAP-IV) scores	[124]
Parkinson’s disease						
*L. acidophilus, B. infantis*	PD patients with GI-NMS	76.05 ± 2.09 years	120	3 months	Improved abdominal pain and bloating compared to the drug trimebutine.	[50]
*L. casei Shirota*	PD patients with chronic constipation	-	40	65 mL of fermented milk containing 6.5 × 10^9^ CFU/ day for 6 weeks.	Improved stool consistency and bowel habits	[51]
Fermented milk with prebiotic fibre (*L. rhamnosus GG, L. acidophilus, L. plantarum, L. paracasei, L. delbrueckii subsp. Bulgaricus, Bifidobacterium*, prebiotic fibre)	PD patients with ROME III criteria constipation	Test: 71.8 ± 7.7 years; placebo: 69.5 ± 10.3 years	120	125 mL fermented milk containing 250 × 10^9^ CFU/day for 4 weeks	Improved frequency of bowel movements	[52]
*L. salivarius* LS01, *L. plantarum* LP01, *L. acidophilus* LA02, *L. rhamnosus* LR06, *B. breve* BR03, *B. animalis* subsp. *lactis* BS01	PBMCs (isolated from PD patients)	70 ± 8 years	-	1 × 10^6^ cells/plate of PBMCs treated with probiotic strains in a 1:1 ratio for 24 h	Decreased the pro-inflammatory cytokines (TNF-α, IL-17A, and IL-6) and oxidative stress levels. Reduced the growth of pathogens such as *E. coli, Klebsiella pneumoniae*	[53]
*L. acidophilus, B. bifidum, L. reuteri, L. fermentum*	PD patients	50 to 80 years	50	2 × 10^9^ CFU per strain; 8 × 10^9^ CFU per capsule; one capsule per day for 12 weeks.	Improved expression of IL-1, IL-8, TNF-α, TGF-β, and PPAR-γ. No changes were observed in inflammation and oxidative stress marker	[54]
*S. thermophilus* DSM 32245, *B. lactis* DSM 32246, *B. lactis* DSM 32247, *L. acidophilus* DSM 32241, *L. helviticus* DSM 32242, *L. paracasei* DSN 32243, *L. plantarum* DSM 32244, *L. brevis* DSM 27961	Male C57BL/6 mice, SH-SY5Y cell line.	9 weeks	30	One sachet containing 200 billion bacteria dissolved in 10 mL of drinking water. Mice received 270 µL using oral gavage daily for 2 weeks	SLAB51^®^ was able to counteract the detrimental effect of 6-OHDA. Improved anti-inflammatory and antioxidant activities. Protected dopaminergic neurons and improved behavioural impairments.	[55]
The commercial probiotic mixture contained *L. rhamnosus* GG, *B. animalis lactis, L. acidophilus*	MPTP-induced male C57BL/6 mice PD model	7 weeks old	-	2 × 10^6^ CFU per day for 30 days	Induced the butyrate synthesis and increased the BDNF and GDNF levels Enhanced the survival and proliferation of dopaminergic neurons. Reduced the expression of monoamine oxidase B expression and increased the expression of neurotrophic factors.	[56]
Hexbio^®^ (*L. acidophilus, L. casei, L. lactis, B. infantis, B. longum*) ProbioM8 *(Bifidobacterium animalis* subsp. *lactis)*	PD patients with ROME III criteria constipation PD patients	50 to 80 years 69.41 ± 6.05 years	48 Probiotic (*n* = 50) Probiotic + conventional drug (*n* = 50)	107 mg of each strain (30 × 10^9^ CFU), 2% FOS and lactose/ twice daily for 8 weeks 2G Probio M8 contains 3 × 10^10^ CFU/day; maltodextrin as an excipient, once daily for 3 months.	Improved bowel opening frequency and whole gut transit time Reduced anxiety and depression. Regulate gut lipid metabolism, SCFAs, and neurotransmitters. Increased dopaminergic synthesis	[125] [58]
Alzheimer’s disease						
*L. acidophilus, L. casei,* *L. fermentum, B. bifidum,*	AD patients	60 to 95 years	60	200 mL probiotic milk containing 2 × 10^9^ CFU/g of each strain per day for 12 weeks.	Improved MMSE score. Decreased MDA and serum hs-CRP	[65]
*L. acidophilus, B. bifidum, B. longum* with selenium	AD patients	55 to 100 years	79	200 µg selenium and probiotic containing 2 × 10^9^ CFU of each strain per day for 12 weeks	Improved cognitive function. Reduced serum triglycerides, LDL, total-/HDL cholesterol, hs-CRP	[66]
Iranian Prodigest Capsule composed of *L. acidophilus*, *B. bifidum* and *B. longum*	Aβ injected Male Sprague-Dawley rats.	-	40	15 × 10^9^ in 1 mL drinking water for 4 weeks and given via ICV for 4 weeks	Improved learning. Increased paired-pulse facilitation ratios. Reduced serum triglycerides and very LDL	[69]
*L. reuteri, L. rhamnosus, B. infantis*	Aβ injected male Wistar rats.	-	50	1 × 10^10^ CFU per day for 10 weeks	Significantly improved spatial memory Reduced Aβ plaques in AD rats. Reduced inflammatory markers IL-1β and TNF-α, and oxidative stress in AD rats.	[70]
*Clostridium butyricum* WZMC1016	APPswe/PS1dE9 Tg mice	2 months	20	1 × 10^9^ CFU per day for 4 weeks	Attenuated microglia-mediated neuroinflammation	[72]
*L. acidophilus* (1688FL431-16LA02), *L. fermentum* (ME3), *B. lactis* (1195SL609-16BS01), *B. longum* (1152SL593-16BL03)	Aβ injected rats	-	60	1 × 10^10^ CFU each strain per day in drinking water (30 mL) every morning.	Improved memory deficit and inhibited AD pathology by modifying microbiota	[73]
*B. breve* A1	Aβ injected mice	10 weeks		1× 10^9^ CFU	Improved cognitive function and ameliorated behaviour deficits. Suppressed the immune reactive and inflammation genes in the hippocampus. Significantly enhanced acetate level	[74]
*L. plantarum* MTCC1325	D-Galactose-induced AD albino rats	-	48	D-Galactose and probiotics 12 × 10^8^ CFU/mL; 10 mL/kg body weight per day for 60 days	Ameliorated cognitive deficits and restored Ach Produced anti-Alzheimer properties against D-Galactose-induced AD rats. Restored spatial memory impairment	[126]
Depression, anxiety and stress						
*B. longum* 1714, and *B. breve* 1205	Innately anxious BALB/c mice	7 weeks	-	Probiotics reconstituted in PBS; 1 × 10^9^ CFU per day for 6 weeks	Reduced anxiety, stress and depression-related behaviours and weight loss in the anxious mouse Reduced stress-induced hyperthermia	[90]
Fermented milk containing Actimel^®^ (*L. delbrueckii* subsp. *bulgaricus*, *S. salivarius* subsp. *thermophilus, L. casei* DN114001)	Healthy students under examination stress	18 to 23 years	155	200 mL fermented milk containing 1 × 10^7^/mL CFU *L. delbrueckii* subsp. *bulgaricus*, 1 × 10^8^ CFU/mL *S. salivarius* subsp. *thermophilus*, and *L. casei* DN114001 per day for 6 weeks	Significantly increased circulating lymphocytes and CD 56 immune cells	[92]
*L. plantarum* P-8	Stressed adults	-	Probiotic (*n* = 43) Placebo (*n* = 36)	2 × 10^10^ CFU per day for 12 weeks	Improved the production of neurotransmitters and neuroactive metabolites. Modulates the gut microbes. Reduced stress and anxiolytic effects	[97]
*B. longum* NCC3001	IBS patients with mild to moderate anxiety and depression scores	-	44	Probiotic sachet containing 1.0 × 10^10^ CFU/g and maltodextrin dissolved in 100–200 mL lactose-free milk per day for 6 weeks	Decreased HAD scores and depression levels significantly. Reduced responses to negative stimuli	[98]
Probiotic capsule containing *L. acidophilus, L. casei* and *B. longum*	Patients with MDD	20 to 55 years	40	2 × 10^9^ CFU per day for 8 weeks	Decreased Beck’s depression scores significantly Significantly raised plasma total glutathione levels	[99]
*L. acidophilus* Rosell-52, *B. longum* Rosell-175	Healthy human volunteers with symptoms of stress	18 to 60 years	75	3 × 10^9^ CFU each strain per day for 3 weeks	No significant changes were observed in psychological symptoms and sleep problems. Reduced stress-induced GI symptoms such as abdominal pain, nausea, and vomiting	[127]
A multivitamin capsule containing probiotics *L. acidophilus, B. bifidum, B. longum*.	Adult men and women suffered from stress and exhaustion.	-	42	1 g capsule containing multivitamins and 10 million strains per day for 6 months	Improved immune health and GI health	[128]
Synbiotics supplement contained *L. helveticus* R0052, *B. longum* R0175, *L. rhamnosus* R0011, galactooligosaccharide and other nutrients.	Healthy stressed individuals	-	32	For 4 weeks	Increased beneficial bacteria load *Lactobacillus* and *Bifidobacterium*. Increased the psychological indices significantly for both positive and negative mood states	[129]
Ecologic ^®^ Barrier containing *B. bifidum* W23, *B. lactis* W51, W52, *L. acidophilus* W37, *L. brevis* W63, *L. casei* W56, *L. salivarius* W24, *L. lactis* W19 and *L. lactis* W58.	Participants with mild to severe depression	18 and above years	71	1 × 10^10^ CFU per day for 8 weeks	Improved cognitive reactivity. No significant differences were found between groups in the BDI, DASS and BAI scores	[130]

* C57BL/6J mice were administered probiotics during a timed mating procedure, and pups separated on the 21st day after the weaning period. CFU: Colony forming unit; MCI: Mild cognitive impairment; ASD: Autism spectrum disorder; MIA: Maternal immune activation: PV+: parvalbumin positive neurons; GABA: Gamma-aminobutyric acid; PBS: Phosphate buffer saline; BDNF: Brain derived neurotrophic factor: ICR: Institute of cancer research; BV2 microglial cell lines: microglial cell derived from C57/BL6 murine; LPS: Lipo polysaccharide; SOD: Super oxide dismutase; GSH: Glutathione; GP: Glutathione peroxidase; MDA: Malondialdehyde; AchE: Acetylcholine esterase; GI: Gastrointestinal; TNF-α: Tumour necrosis factor α; PD: Parkinson’s disease; GI-NMS: Gastrointestinal Non-motor symptoms; PBMCs: Peripheral blood mononuclear cells; IL-17A: Interleukin 17A; IL6: Interleukin6; IL: 1-Interleukin1; IL-8: Interleukin 8; IL1β: Interleukin 1β; TGF-β: Tumour growth factor-β; PPAR-γ: Peroxisome proliferator-activated receptor gamma; C57BL/6J: C57 Black 6 Jackson laboratory strain mice; SH-SY5Y cells: Thrice sub-cloned cell line from SK-N-SH neuroblastoma cells; 6-OHDA: 6-hydroxydopamine; MPTP: 1-Methyl-4-phenyl-1,2,3,6-tetrahydropyridine; GDNF: Glial cell-line derived neurotrophic factor; AD: Alzheimer’s disease; MMSE: Mini-Mental State Examination; hs-CRP- High sensitivity C-reactive protein; LDL: Low density lipoprotein; HDL: High density lipoprotein; ICV: Intracerebroventricular; Aβ: Amyloid beta; APPswe/PS1DE9 Tg: Two transgenes with AD mutation with a chimeric mouse and human amyloid beta precursor protein with Swedish mutation and human presenilin-1 (PS-1) lacking exon 9 (dE9), under the mouse prion protein promoter containing transgenic mice. Ach: Acetylcholine; BALB/c mice: Albino laboratory-bred strain of house mouse; CD56: Cluster of differentiation 56; IBS: Irritable bowel syndrome; HAD: Hospital anxiety depression scale; MDD: Major depressive disorder; BDI: Beck depression index; BAI: Beck anxiety inventory; DASS: Depression anxiety stress scale.

## Data Availability

Not applicable.
